# Sex-based differences in the prevalence of acute mountain sickness: a meta-analysis

**DOI:** 10.1186/s40779-019-0228-3

**Published:** 2019-12-09

**Authors:** Yun-Peng Hou, Jia-Lin Wu, Chao Tan, Yu Chen, Rui Guo, Yong-Jun Luo

**Affiliations:** Department of Military Medical Geography, Army Medical Service Training Base, Army Medical University, Chongqing, 400038 China

**Keywords:** Prevalence, Sex differences, Acute mountain sickness, Risk factors

## Abstract

**Background:**

When lowlanders rapidly ascend to altitudes > 2500 m, they may develop acute mountain sickness (AMS). The individual susceptibility, ascending velocity, time spent at altitude, activity levels and altitude reached are considered risk factors for AMS. However, it is not clear whether sex is a risk factor. The results have been inconclusive. We conducted a meta-analysis to test whether there were sex-based differences in the prevalence of AMS using Lake Louise Scoring System.

**Methods:**

Systematic searches were performed in August 2019 in EMBASE, PubMed, and Web of Science for prospective studies with AMS data for men and women. The titles and abstracts were independently checked in the primary screening step, and the selected full-text articles were independently assessed in the secondary screening step by the two authors (YPH and JLW) based on pre-defined inclusion criteria. The meta-analysis was performed using by the STATA 14.1 software program. A random-effects model was employed.

**Results:**

Eighteen eligible prospective studies were included. A total of 7669 participants (2639 [34.4%] women) were tested. The results showed that there was a statistically significant higher prevalence rate of AMS in women than in men (*RR* = 1.24, 95%CI 1.09–1.41), regardless of age or race. Howerver, the heterogeneity was significant in the analysis (Tau^2^ = 0.0403, Chi^2^ = 50.15, *df* = 17; *I*^2^ = 66.1%, *P* = 0.000), it was main caused by different numbers of subjects among the studies (coefficient = − 2.17, *P* = 0.049). Besides, the results showed that there was no evidence of significant publication bias in the combined studies on the basis of Egger’s test (bias coefficient = 1.48, *P* = 0.052) and Begg’s test (*P* = 0.130).

**Conclusions:**

According to this study, the statistically significant finding emerging from this study was that women have a higher prevalence of AMS. However, the authors could not exclude studies where patients were on acetazolamide. Our analysis provided a direction for future studies of the relationship of sex and the risk of AMS, such as the pathological mechanism and prevention research.

## Background

Acute mountain sickness (AMS) may occur when a person who is used to being at a low altitude ascends to a higher altitude [1]. The typical symptoms include headache, anorexia, nausea, vomiting, dyspnoea, lassitude, and insomnia after arriving at a high altitude. This condition is termed AMS. It is a clinical syndrome in which the body decompensates in response to acute hypoxic conditions [2–4]; AMS is exacerbated by exercise and can be disabling [5]. More seriously, if symptoms are ignored, AMS can develop into life-threatening high-altitude cerebral edema [6]. The individual susceptibility, ascending velocity, time spent at altitude, activity levels and altitude reached may be the common causes of AMS [7]; men and women present with different AMS morbidity profiles. Previous studies that reported sex as a risk factor for AMS were inconsistent, although some indicated that women are more likely to suffer from AMS than men. For example, in Murdoch’s report, the prevalence of AMS was 88.6% vs 69.0% (women vs. men, respectively) [4], and rates of 60.0% vs. 21.9% (women vs. men, respectively) were reported in the study by other authors [8], while other studies showed a higher prevalence in men [9, 10] or no sex-based difference [11, 12]. Although it has been suggested that sex-based differences in the prevalence of AMS patients exist, to date, no systematic review or meta-analysis has addressed this issue.

The perspective in the existing literature is that the differences between men and women are mainly determined by the physical differences and the different hormone levels [13, 14]. Some investigators believe that the differences in the prevalence of AMS between men and women is also affected by hormones or other factors associated with hormones [15]. However, that is only hypothesis, and the pathophysiological mechanism of AMS is still not entirely clear. To determine whether there are sex-based differences in the prevalence of AMS, we conducted a systematic literature review of studies using the same criteria and performed a meta-analysis to quantify the results.

## Methods

This review was conducted according to the PRISMA (Preferred Reporting Items for Systematic Reviews and Meta-analyses) guidelines [16].

### Search strategy

Searches were conducted in PubMed, EMBASE and Web of Science for articles published before August 2019. The search strings included terms pertaining to: 1) AMS (such as, acute mountain sickness, acute high altitude disease, acute mountain illness, altitude disease, Lake Louise Scoring System (LLSS)); 2) epidemiological indicators of disease (such as, prevalence, incidence, risk, epidemiology); and 3) subjects characteristics (such as, sex, gender), using the logical connectives “OR” and “AND” to combine them. The titles and abstracts of the returned articles were searched for the relevant variables, and the initial eliminations were made. The publication dates were limited to article published after 1991 because the LLSS was first propounded in February, 1991 [17]. The language was restricted to English. Furthermore, the studies listed in the references of the articles were reviewed.

### Study selection

Two authors, YPH and JLW, independently reviewed the publications. We first applied Endnote X9 software to eliminate duplicate publications, and read the titles and abstracts to initially select candidate articles. For those publications that were not clearly described, we screened them by downloading and reading the full texts, and discrepancies were resolved by consensus. The eligible studies met the following criteria:

1) The studies were limited to prospective studies with high reliability and sufficient data. Clinical research, interventional experiments or retrospective studies were excluded due to the possibility of selection bias.

2) In terms of the diagnostic criteria, the included studies adopted the same data collection technology, used the LLSS [17], and applied the same two cut-off values (LLSS ≥3 or ≥ 4) to define AMS. Studies using other diagnostic criteria were excluded from the pooled analysis because diverse criteria may result in different prevalence, affecting the sex-based differences.

3) The studies included sex-specific numbers or rates, or the data needed to calculate the same, i.e., the prevalence or percentages of men and women with AMS.

4) The average age of the subjects was over 18 years, as younger subjects are not sufficiently physically mature to enable the assessment of sex-based effects.

5) The minimum altitude was 2500 m. This height can cause physical changes, such as acute altitude sickness, high altitude pulmonary edema and other diseases.

### Data extraction

The data extraction table was developed by YPH and JLW. Disagreements were reconciled through consensus in face-to-face meetings, and consensus was reached after discussion.

The information extracted from each study included the first author, publication year, location, average age, race, participant type, altitude, cut-off value for the LLSS to identify AMS, and number of women or men with AMS or the AMS prevalence rates.

### Assessment of AMS

The methods for the assessment of AMS include the LLSS, the Environmental Symptoms Questionnaire III (ESQ-III) and so on [18, 19]. All of these methods are widely utilized in studies of the effects of altitude, but there is still no golden standard and the methods for the assessment of AMS depend on subjective symptoms. Some articles have compared the LLSS with the ESQ-III AMS score, subjects are likely to receive a different AMS diagnosis when evaluated by different scoring systems [20, 21]. Despite that, this meta-analysis was performed based on the LLSS. This criterion aimed to reduce the confounding factors introduced by means of different evaluation methods and improve the quality of the assessment. Scores in the LLSS range from 0 to 12, and a total score ≥ 3 in the presence of a headache was the diagnostic criterion for AMS. However, some researchers used 4 points as a cut-off value to diagnose AMS [22, 23]. We therefore concluded that a subgroup analysis was needed to evaluate the implications of the different cut-off values.

### Quality assessment

The methodological quality of each study using LLSS as assessed based on the tool developed by Loney et al. [24], which aimed to critically appraise research articles that estimate the prevalence or incidence of a disease. Two authors (YPH and JLW) independently implemented this method, with all disagreements resolved by consensus. The scoring system is an 8-point scale consisting of three parts: validity of research methods (0–6 points), interpretation of the results (0–1 point) and applicability of the results (0–1 point). Detailed scores for each study can be found in Appendix. A total score of 4 or 5 is considered adequate quality, and a score ≥ 6 points is defined as high quality. However, for publications with a score ≤ 3 were excluded to ensure that the included studies had adequate reliability and methodological quality.

### Statistical analysis

The meta-analysis was performed using Stata 14.1 (Stata Corp, College Station, TX, USA). We used a random-effects model to aggregate the data because the random-effects model is more conservative than the fixed-effect model; in addition, it allows for the existence of heterogeneity. Relative risks (*RR*) were used to assess the binary outcomes variables rather than odds ratios (*OR*), as the RR are easier to explain and do not overestimate the magnitude of the effect [25]. Heterogeneity among studies was tested using the I2 statistic. Meta-regression analysis and subgroup analysis were used to verify the source of the heterogeneity. Egger’s test, Begg’s test and meta-funnel plot asymmetry were used to test for the presence of publication bias [26]. There is a significant difference if *P* < 0.05.

## Results

### Search results

**Fig. 1:**
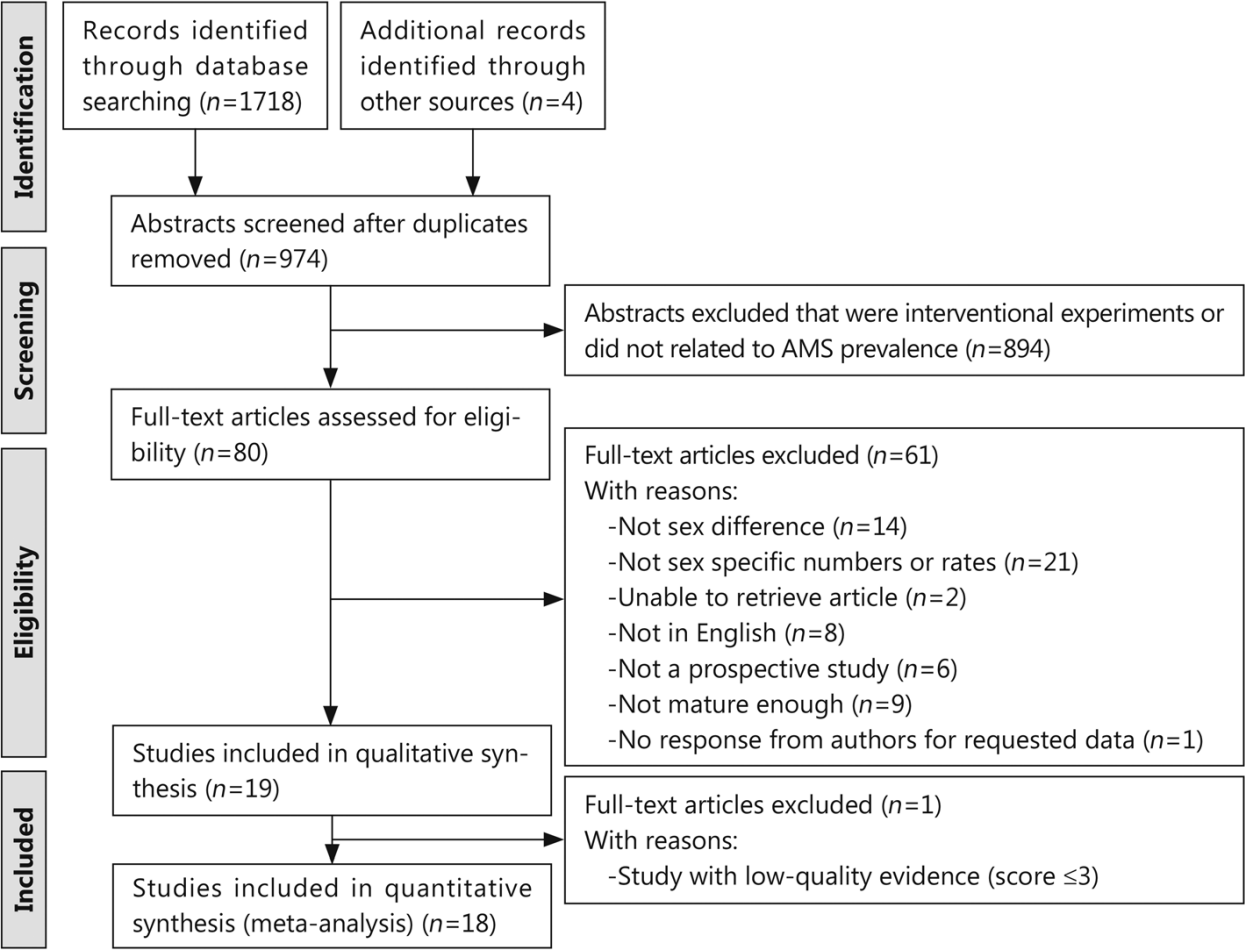
Flow chart of the study selection process. The flowchart describes the process of searching for and screening of eligible studies on AMS

A total of 1718 publications relevant to AMS were identified in the databases. Additionally, 4 additional records were identified through other sources. The abstracts of 974 were reviewed, of which 80 articles were reviewed in full, and 18 were ultimately included. The excluded studies were thirty-one with no sex-based data reported or specific numbers, eight that were not in English, six that were not prospective studies, fourteen with no sex differences, nine with average ages < 18 years, two without full-text versions available and one without a response from the authors regarding requested data. Therefore, a total of 18 full-text articles were included in this meta-analysis (Table 1), and the selection flow chart is shown in Fig. 1.

**Table 1:** Details of eligible AMS-related studies that were included in the meta-analysis

References	Location	Race of subjects	Subjects	Diagnostic criteria	Average age (years)	Altitude (m)	Total subjects (*n*)	Prevalence (%)		
								Total	Women	Men
Murdoch et al. (1995) [4]	Shyangboche, Asia	Asian	Guests	LLSS ≥3	45.3	3740	154	77.9 (120/154)	88.6 (62/70)	69.0 (58/84)
Ziaee et al. (2003) [9]	Mount Damavand, Asia	Asian	Hikers	LLSS ≥3	31.9	4200	459	60.8 (279/459)	58.1 (86/148)	63.1 (196/311)
Wagner et al. (2008) [10]	Mt. Whitney, North America	American	Hikers	LLSS ≥3	37.6	4419	886	42.6 (337/886)	37.7 (80/212)	44.1 (297/674)
Jafarian et al. (2008) [27]	Tehran, Asia	Asian	Volunteers	LLSS ≥3	28.8	3450	90	37.8 (34/90)	53.3 (16/30)	30.0 (18/60)
Mairer et al. (2009) [22]	Austrian Alps, Europe	European	Hikers	LLSS ≥4	37.4	2200–3500	422	16.6 (70/422)	18.9 (20/106)	15.8 (50/316)
Wu et al. (2010) [28]	Lhasa, Asia	Asian	Passengers	LLSS ≥3	40.4	2600–5072	222	27.0 (60/222)	34.5 (30/87)	22.2 (30/135)
Wang et al. (2010) [11]	Jade Mountain, Asia	Asian	Hikers	LLSS ≥3	40.2	3925	1066	36.0 (384/1066)	36.3 (128/353)	35.9 (256/713)
Mairer et al. (2010) [29]	Alps, Europe	European	Mountaineers	LLSS ≥4	34.7(group 1) 36.8(group 2)	3454 and 3817	155	37.4 (58/155)	39.3 (11/28)	37.0 (47/127)
Modesti et al. (2011) [8]	Mount Everest Base Camp, Asia	Asian	Volunteers	LLSS ≥4	40	5400	47	34.0 (16/47)	60.0 (9/15)	21.9 (7/32)
Chen et al. (2012) [23]	Jade Mountain, Asia	Asian	Hikers	LLSS ≥4	42	3402–3952	787	32.8 (258/787)	34.7 (92/265)	31.8 (166/522)
Maclnnis et al. (2013) [30]	Gosainkunda,Asia	Asian	Pilgrims	LLSS ≥3	36.7	4380	491	34.0 (167/491)	45.5 (67/147)	29.1 (100/344)
Mandolesi et al. (2014) [31]	Mount Rosa, Europe	European	Mountaineers	LLSS ≥3	36.4	3647–4559	60	40.0 (24/60)	54.5 (6/11)	36.7 (18/49)
Hsu et al. (2015) [32]	Jiaming Lake,Asia	Asian	Mountaineers	LLSS ≥3	19.8	3550	91	20.9 (19/91)	14.3 (4/28)	23.8 (15/63)
Ren et al.(2015) [33]	Lhasa, Asia	Asian	Volunteers	LLSS ≥4	38.4	3100–4300	80	43.8 (35/80)	53.1 (26/49)	29.0 (9/31)
Horiuchi et al. (2016) [34]	Mount Fuji,Asia	Asian	Climbers	LLSS ≥3	36.1	3776	345	29.5 (98/345)	32.6 (46/141)	25.5 (52/204)
Sánchez-Mascuñano et al. (2017) [35]	Spain, Europe	European	Travellers	LLSS ≥3	37.7	> 3400	302	25.8 (78/302)	39.0 (53/156)	17.1 (25/146)
Horiuchi et al. (2018) [12]	Mount Fuji,Asia	Asian	Climbers	LLSS ≥3	37.4	> 2870	1932	31.6 (610/1932)	32.9 (252/767)	30.7 (358/1165)
J. Boos et al. (2018) [36]	Himalayas,Asia	Asian	Military servicemen	LLSS ≥3	32.1	5140	80	47.5 (38/80)	69.2 (18/26)	37.0% (20/54)

*AMS* acute mountain sickness, *LLSS* Lake Louise Scoring System

### Selected studies and characteristics

A total of 18 studies [4, 8–12, 22, 23, 27–36] on AMS using LLSS were included in this analysis, and the detailed information is shown in Table 1. The publication period ranged from 1995 to 2018, with the majority of the publication dates being after 2000. The experimental subjects included guests, pilgrims, hikers, volunteers, and mountaineers. The study altitudes ranged from 2200 m to 5400 m, but the altitude in most studies was above 2500 m. The number of subjects was between 47 and 1932, and the total number included in the analysis was 7669. The highest overall prevalence of AMS was 77.9%, and the lowest was 16.6% [4, 22]. The maximal single-study prevalence rates for AMS in women and men were 88.6 and 69.0%, respectively, whereas the minimal values in women and men were 14.3 and 15.8%, respectively [4, 22, 32]. Fifteen studies reported that women had a higher prevalence of AMS than men in the same experiment. It should be noted that all of the studies used the LLSS for the diagnosis of AMS, but 4 of them defined the diagnostic criterion as an LLSS value of at least 4 with headache present, whereas the remaining 15 studies defined the criterion as an LLSS score of at least 3 with headache present. In selecting the studies, some studies were excluded on the basis of ambiguous data regarding the number of subjects or the prevalence despite demonstrating a sex-based distinction [37].

### Quality assessment

The details of the quality assessment of the included studies are listed in Appendix; 4 studies were rated “high quality” (22.2%, total score ≥ 6), 14 studies were considered “good quality” (77.8%; total score = 4 to 5), and there was one thesis rated “low quality” (total score ≤ 3). The limitations affecting the quality of the studies were generally the following: small sample size (10 of 18 studies), refusal to participate not described (16 of 18 studies), biased assessors (17 of 18 studies) and 95% confidence intervals not provided (13 of 18 studies). To ensure the reliability of the included studies, we excluded low-quality studies, and 18 studies were included in the final meta-analysis.

### Meta-analysis results of sex-based difference in AMS

**Fig. 2:**
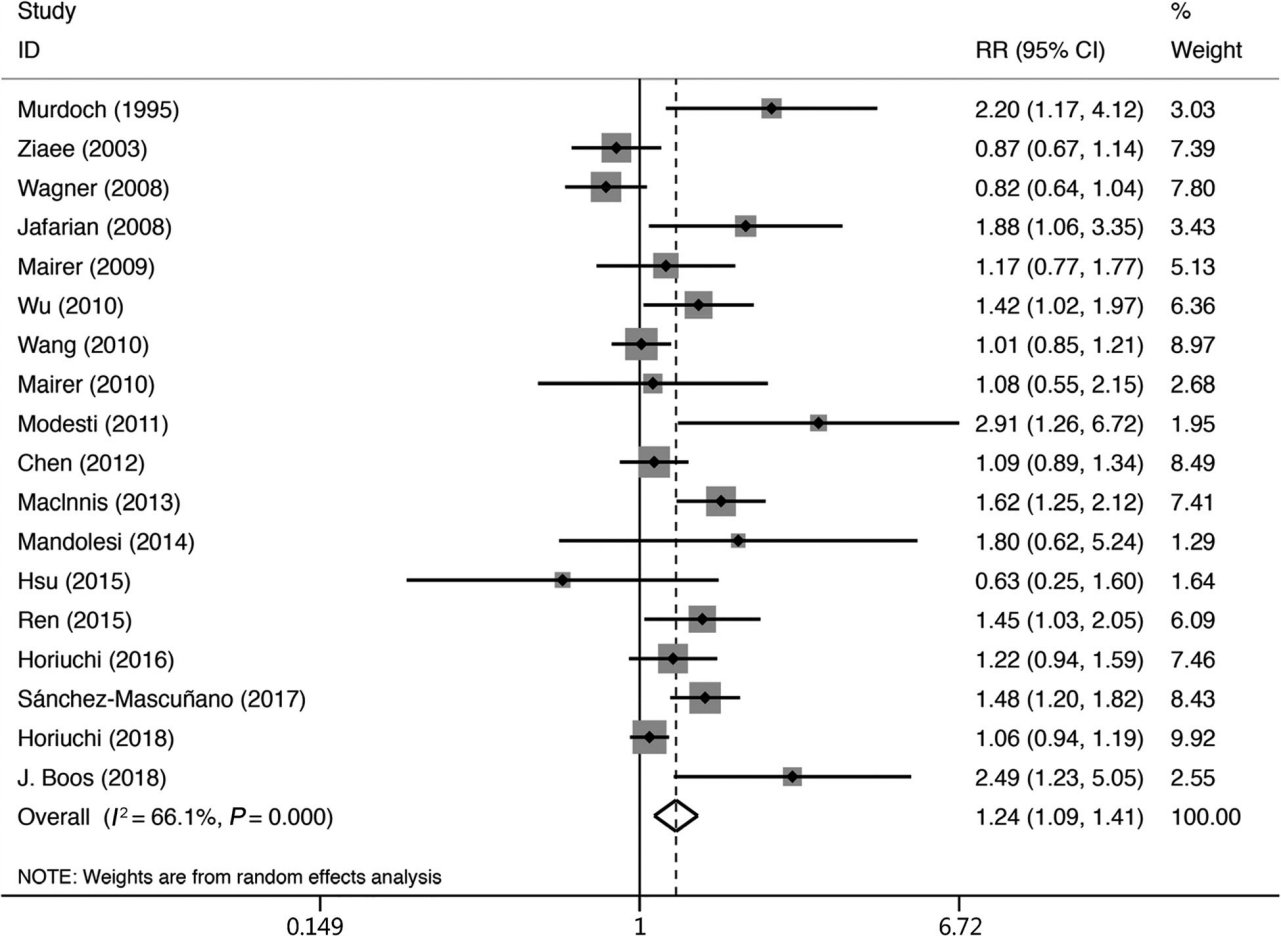
Forest plot of the 18 AMS studies in the random-effects model. Summaries of the men and women results for the risk of AMS are displayed. The risk factors are indicated based on the relative risk for women with regard to the prevalence of AMS.Heterogeneity: Tau^2^ = 0.0403, Chi^2^ = 50.15, *df* = 17; *I*^2^ = 66.1%, *P* = 0.000. Test for overall effect: *Z* = 3.29, *P* = 0.001. *RR*: relative risk; 95% CI: 95% confidence interval

We selected a fixed-effects model for the initial stage of the analysis, but the heterogeneity did not meet the condition for this model (Tau^2^ = 0.0403, Chi^2^ = 50.15, *df* = 17; *I*^2^ = 66.1%, *P* = 0.000). We therefore chose a random-effects model for the final evaluation of the data. The results showed that there was a statistically significant higher prevalence of AMS in women than in men (*RR* = 1.24, 95% CI 1.09–1.41). The *RR* values for the individual studies and the pooled estimate are shown in Fig. 2.

### Meta-regression analysis

The heterogeneity was significant in the analysis (*I*^2^ = 66.1%, *P* = 0.000), so we performed the meta-regression analysis to explore the contribution of the four covariates (race, age, LLSS cut-off value and number of subjects) in the heterogeneity. The results indicated that the number of subjects was a possible contributor to the heterogeneity (coefficient = − 2.17, *P* = 0.049). The contributions of race, LLSS cut-off value and age were not obvious (*P* = 0.826, *P* = 0.901, *P* = 0.970, respectively, Table 2).

**Table 2:** Covariates in the meta-regression analysis of AMS studies

Heterogeneous factors	Coefficient	Standard error	*t*	*P*
Race of subjects	−0.0415275	0.1847339	−0.22	0.826
Number of subjects	−0.3564554	0.1640766	−2.17	0.049
LLSS cut-off value	−0.0223583	0.1768234	−0.13	0.901
Average age	0.0068726	0.1774487	0.04	0.970
Constant	0.8983218	0.4944899	1.82	0.092

*LLSS* Lake Louise Scoring System

### Subgroup analysis

The result of the regression analysis showed that different numbers of subjects (*n* < 300 vs. *n* ≥ 300) was the main cause of the heterogeneity, and the heterogeneity was improved after the subgroup analysis (*I*^2^ = 24.6%, *P* = 0.225). The evaluation of the effect of the number of subjects showed that the studies with small sample sizes had a higher rate of AMS (*RR* = 1.60, 95% CI 1.27–2.00) compared with those that with larger sample sizes (*RR* = 1.12, 95% CI 0.98–1.28).

Three other subgroups (race, age, LLSS cut-off value) were analyzed in the context of the overall estimate by means of different stratifications. Subgroup analyses were performed to determine whether sex-based differences emerged in subgroups stratified by race, but the researchers found no statistically significant differences between Asian and non-Asian populations (*RR* = 1.27 vs. *RR* = 1.16), indicating that people of different races have similar susceptibilities to AMS.

Moreover, the results for other subgroups showed that there was no evidence that sex-based differences were affected by age (average age < 40 years vs. ≥40 years) or LLSS cut-off value (LLSS ≥3 vs. ≥4), and all subgroup analysis data are shown in Table 3.

**Table 3:** The heterogeneity of the subgroup analysis of the included AMS studies

Subgroup	Subjects number		AMS [*n*(%)]		*RR* (95%CI)	*P*
	Women	Men	Women	Men		
All participants	2639	5030	1006(38.1)	1722(34.2)	1.24(1.09–1.41)	0.000
Race						
Aisan	2126	3718	836(39.3)	1285(34.6)	1.27(1.10–1.48)	0.000
No-Aisan	513	1312	170(33.1)	437(33.3)	1.16(1.09–1.41)	0.005
Number of subjects						
<300	344	635	182(52.9)	222(35.0)	1.60(1.27–2.00)	0.225
≥ 300	2295	4385	824(35.9)	1500(34.2)	1.12(0.98–1.28)	0.001
LLSS cut-off value						
≥ 3	2176	4002	848(39.0)	1443(36.1)	1.25(1.08–1.45)	0.000
≥ 4	463	1028	158(34.1)	279(27.1)	1.27(1.01–1.60)	0.168
Average age						
<40 years	1849	3544	685(37.0)	1205(34.0)	1.32(1.02–1.71)	0.000
≥ 40 years	790	1486	321(40.6)	517(34.8)	1.22(1.04–1.44)	0.013

*LLSS* Lake Louise Scoring System, *AMS* acute mountain sickness; *RR* relative risk, *95% CI* 95% confidence interval

### Publication bias

**Fig. 3:**
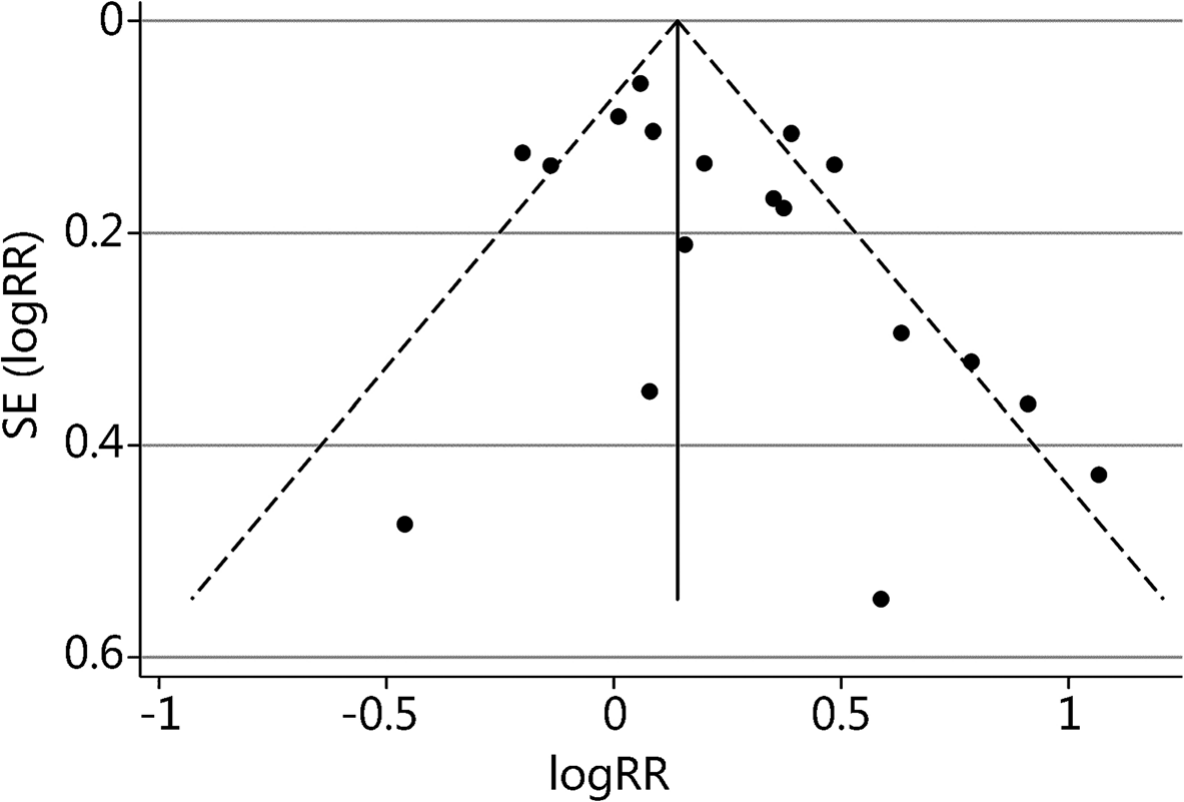
Funnel plot of the 18 AMS studies to assess publication b.ias. Note the symmetrical distribution of the studies. In addition, all studies were combined and subjected to Egger’s test (bias coefficient = 1.48, *P* = 0.052) and Begg’s test (*P* = 0.130). LogRR: log relative risk; SE (LogRR): standard error of the log relative risk

Publication bias was assessed with meta-funnel plots (Fig. 3), Egger’s test and Begg’s test. The results showed that there was no evidence of significant publication bias in the combined studies on the basis of Egger’s test (bias coefficient = 1.48, *P* = 0.052) and Begg’s test (*P* = 0.130).

## Discussion

The main purpose of this meta-analysis was to evaluate whether there is a difference between women and men in terms of their susceptibility to AMS using LLSS. After excluding the studies that did not meet the screening criteria, a total of 18 studies were included in this systematic meta-analysis. The results showed that the prevalence of AMS is approximately 1.24 times greater in women than in men, regardless of age or race, however, we could not exclude studies where patients were on acetazolamide. Although no previous systematic evaluation or meta-analysis has shown that AMS has obvious sex-based differences, most of studies are consistent with the results of this meta-analysis (total 15/18); for example, MacInnis et al. [30] reported that the prevalence in women was 45.5%, which was 12.5% higher than the prevalence in men, indicating that women were more likely than men to suffer from AMS (45.5% vs 34.0%, *RR* = 1.62). In contrast, there have been reports that men are more likely than women to suffer from AMS [9, 10].

Many mechanisms can explain the relatively high prevalence in women. One hypothesis regarding the pathogenesis is intracranial hypertension [38, 39]. Two factors contributing to increased intracranial pressure need attention: vascular permeability and fluid retention. Oestrogen is thought to upregulate vascular endothelial growth factor (VEGF) expression [40]. VEGF is responsible for the augmentation of vascular leakage [41], which increases the exudation of tissue fluid and causes intracranial hypertension. Another factor is related to fluid retention. In an early study, the subjects that developed severe AMS displayed water retention within the first 3 h of altitude exposure; healthy subjects, in contrast, exhibited mild diuresis, or the excretion of urine [42]. The study speculated that this rapid effect is due to an early increase in the anti-diuretic hormone (ADH), which is a hormone that is responsible for water re-absorption by the kidneys. Oestrogen has been shown to lower the threshold for ADH, which causes an increase in fluid retention [43]. This provides another potential mechanism explaining the results of this study.

The second mechanism relates to the concentration of erythropoietin (EPO). After exposure to high altitude, blood components associated with oxygen delivery are affected; the concentration of hemoglobin and count of red blood cells increased sharply [44], which are thought to be advantageous compensations [45]. Testosterone is known to be an androgen that promotes erythropoiesis, which may possibly improve oxygen carrying capacity by increasing EPO levels, conferring an advantage on men at high altitudes [46]. The EPO concentration increases within hours of ascent and stimulates a gradual increase in hemoglobin for men at high altitude; at that point, the human body exhibits a hematological adaptation, reducing the prevalence of AMS. Furthermore, this is often exploited by male athletes who train at high altitude to increase the oxygen-carrying capacity of their blood to improve sea-level endurance and performance [47].

However, including studied reporting LLS only may limit a large number of studies. Previous researchers have made comparisons between the ESQ-III and the LLSS, they may identify different populations as suffering from AMS [21, 48]. Wanger et al. [20] found that the criterion of LLSS ≥3 with a headache and at least one additional symptom resulted in 63% of the climbers being diagnosed with AMS, there was a discrepancy in the diagnosis of AMS in about 16% of the cases which ESQ-III was used. Dellasanta et al. [21] found that using a LLSS score of ≥3 labeled more than twice as many persons as suffering from AMS as were identified with a ESQ-III AMS criterion score of ≥0.7. Therefore, pooled studies using LLSS criterion with studies using other criterion in an analysis is not recommended.

Finally, because of time, energy and other objective constraints, the research has certain limitations. First, as mentioned in the previous paragraph, there was significant heterogeneity within this meta-analysis. The meta-regression and subgroup analysis also indicated the presence of heterogeneity, so it was difficult to avoid bias. Second, some variables within the studies used, including the race of the subjects, the number of subjects who used prophylactic drugs before the experiment and others, could not be standardized. These elements were difficult to resolve in the processing of the studies for analysis. For this reason, some of the heterogeneity may have occurred as a result of these differences among the studies. Third, the inclusion criteria were strict; for example, we selected the LLSS score as the only accepted diagnostic criterion and excluded other systems such as the ESQ-III. In addition, studies that were not prospective were also excluded. The aims of applying these criteria were to reduce the heterogeneity and improve the quality of the studies selected.

## Conclusions

According to this study, women are more likely than men to suffer from AMS (*RR* = 1.24, 95% CI 1.09–1.41), but the conspicuous studies’ heterogeneity (*I*^2^ = 66.1%, *P* = 0.000) will reduce the reliability of the conclusion. Our analysis provided a direction for future studies of the relationship of sex and the risk of AMS, such as the pathological mechanism and prevention research.

## Data Availability

All data are fully available without restriction.

## Appendix

**Table 4:** Critical appraisal of 18 studies on the prevalence of AMS

Study and setting	Subjects (*n*)	Sample design	Sampling frame	Measures	Unbiased assessors	Response rate and refusers	Prevalence rate	Score and limitations
Murdoch et al. (1995) [4] Shyangboche, Asia	154	Guests Mean 45.3 years	Guests staying at hotel	Lake Louise consensus AMS self assessment scoring system	Microbiologist Negative screens not assessed	97.5% Refusers not described	89% of famales 69% of males CI given for OR	Score 5 Poor sample size Negative screens not assessed Refusers not described No CI given
Ziaee et al. (2003) [9] Mount Damavand, Asia	459	Trekkers Mean 31.9 years	Trekkers around Mount Damavand in Iran	Lake Louise consensus AMS self assessment scoring system	Pediatrist Epidemiologist Infectious diseases scientist Negative screens not assessed	100% Refusers not described	60.8% (279/459) No CI given	Score 5 Negative screens not assessed Refusers not described No CI given
Wagner et al. (2008) [10] Mount Whitney, North America	886	Hikers Mean 37.6 years	Trekkers on Mt.Whitney	Lake Louise consensus AMS self assessment scoring system	Six interviewers Negative screens not assessed	100% Refusers not described	42.6% (337/886) No CI given	Score 5 Negative screens not assessed Refusers not described No CI given
Jafarian et al. (2008) [27] Tehran, Asia	90	Volunteers Mean 28.8 years	Individuals in a mountain hotel’s clinic	Lake Louise consensus AMS self assessment scoring system	Neurologist Anesthesiologist Negative screens not assessed	100% Refusers not described	37.8% (34/90) CI given for subgroup	Score 5 Poor sample size Negative screens not assessed Refusers not described
Mairer et al. (2009) [22] Austrian Alps, Europe	422	Recreational hikers Mean 37.4 years	Recreational hikers	Lake Louise consensus AMS self assessment scoring system	Medical researcher Negative screens not assessed	90% Refusers described	16.6% (70/422) No CI given	Score 6 Negative screens not assessed No CI given
Wu et al. (2010) [28] Lhasa, Asia	222	Passengers Mean 40.4 years	Qinghai–Tibet railroad passengers	Lake Louise consensus AMS self assessment scoring system	Physiological Research Group of the Ministry of Railroad Negative screens not assessed	100% Refusers not described	27.0% (60/222) No CI given	Score 4 Poor sample size Negative screens not assessed Refusers not described No CI given
Mairer et al. (2010) [29] Alps, Europe	155	Mountaineers Mean 34.7 years (group 1) Mean 36.8 years (group 2)	Trekkers in both the Eastern and Western Alps	Lake Louise consensus AMS self assessment scoring system	Sport Scientists Medical researchers Negative screens not assessed	100% Refusers described	37.4% (58/155) No CI given	Score 5 Poor sample size Negative screens not assessed No CI given
Wang et al. (2010) [11] Jade Mountain, Asia	1066	Hikers Mean 40.2 years	Trekkers visiting Paiyun Lodge on Jade Mountain	Lake Louise consensus AMS self assessment scoring system	Health workers Medical researchers and doctors Negative screens not assessed	100% Refusers not described	36.0% (384/1066) No CI given	Score 5 Negative screens not assessed Refusers not described No CI given
Modesti et al. (2011) [8] Mount Everest Base Camp, Asia	47	Volunteers Mean 40 years	Recruited volunteers	Lake Louise consensus AMS self assessment scoring system	Medical researchers, doctors statistician Negative screens assessed	100% Refusers not described	34.0% (16/47) No CI given	Score 5 Poor sample size Refusers not described No CI given
Chen et al. (2012) [23] Jade Mountain, Asia	787	Random sample Hikers Mean 42 years	Trekkers on Jade Mountain	Lake Louise consensus AMS self assessment scoring system	Doctors, Health Scientists Negative screens not assessed	100% Refusers not described	32.8% (258/787) No CI given	Score 5 Negative screens not assessed Refusers not described No CI given
Maclnnis et al. (2013) [30] Gosainkunda,Asia	491	Pilgrims Mean 36.7 years	Pilgrims travel to the Janai Purnima festival in Gosainkunda, Nepal (4380 m)	Lake Louise consensus AMS self assessment scoring system	medical student or intern Negative screens not assessed	100% Refusers not described	34.0% (167/491) CI given for RR	Score 6 Negative screens not assessed Refusers not described
Mandolesi et al. (2014) [31] Mount Rosa, Europe	60	Mountaineers Mean 36.4 years	Recruited Mountaineers	Lake Louise consensus AMS self assessment scoring system	Medical researchers Negative screens not assessed	100% Refusers not described	40.0% (24/60) No CI given	Score 4 Poor sample size Negative screens not assessed Refusers not described No CI given
Hsu et al. (2015) [32] Jiaming Lake, Asia	91	Mountaineers Mean 19.8 years	Mountaineers climbing to Jiaming Lake in Taiwan	Lake Louise consensus AMS self assessment scoring system	Three trained emergency physicians Negative screens not assessed	100% Refusers not described	20.9% (19/91) No CI given	Score 4 Poor sample size Negative screens not assessed Refusers not described No CI given
Ren et al.(2015) [33] Lhasa, Asia	80	Volunteers Mean 38.4 years	Recruited volunteers	Lake Louise consensus AMS self assessment scoring system	Medical researcher Negative screens not assessed	100% Refusers not described	43.8% (35/80) No CI given	Score 4 Poor sample size Negative screens not assessed Refusers not described No CI given
Horiuchi et al. (2016) [34] Mount Fuji	345	Climbers Mean 36.1 years	Climbers on Mt.Fuji	Lake Louise consensus AMS self assessment scoring system	Medical researchers Negative screens assessed	88.9% Refusers not described	29.5% (98/345) No CI given	Score 5 Poor response rate Refusers not described No CI given
Sánchez-Mascuñano et al. (2017) [35] Spain, Europe	302	Travellers Mean 37.7 years	Travellers in Barcelona,Spain	Lake Louise consensus AMS self assessment scoring system	Trained medical doctor Negative screens not assessed	92.4% Refusers described	25.8% (78/302) 95% CI 20.9–30.8	Score 7 Negative screens not assessed
Horiuchi et al. (2018) [12] Mount Fuji, Asia	1932	Climbers Mean 37.4 years	Climbers on Mt.Fuji in Japan	Lake Louise consensus AMS self assessment scoring system	Scientific researcher Negative screens not assessed	100% Refusers not described	31.6% (610/1932) CI given for subgroup	Score 6 Negative screens not assessed Refusers not described
J. Boos et al. (2018) [36] Himalayas, Asia	80	Military servicemen Mean 32.1 years	Dhaulagiri region of the Himalayas	Lake Louise consensus AMS self assessment scoring system AMS-C Scores State-Trait-Anxiety-Score	Medical researchers Negative screens not assessed	100% Refusers not described	47.5% (38/80) CI given for Independent predictors	Score 5 Poor sample size Negative screens not assessed Refusers not described

*AMS* acute mountain sickness, *CI* confidence interval, *RR* relative risks, *OR* odds ratios. The critical appraisal was used to estimate the quality of the published articles, determine the validity and usefulness of prospective studies and improve the overall quality of the included articles

## Abbreviations

ADH: Anti-diuretic hormone
AMS: Acute mountain sickness; 95% CI: 95% confidence interval
EPO: Erythropoietin
ESQ-III: Environmental Symptoms Questionnaire III
LLSS: Lake Louise Scoring System
*OR*: Odds ratio
*RR*: Relative ratio
VEGF: Vascular endothelial growth factor
